# Novel Antioxidant Peptides from *Grateloupia livida* Hydrolysates: Purification and Identification

**DOI:** 10.3390/foods11101498

**Published:** 2022-05-20

**Authors:** Xiao Hu, Chuang Pan, Miaomiao Cai, Laihao Li, Xianqing Yang, Huan Xiang, Shengjun Chen

**Affiliations:** 1 Key Laboratory of Aquatic Product Processing, Ministry of Agriculture and Rural, South China Sea Fisheries Research Institute, Chinese Academy of Fishery Sciences, Guangzhou 510300, China; hnhuxiao@163.com (X.H.); silverpfoxc@hotmail.com (C.P.); mm_cai960319@163.com (M.C.); laihaoli@163.com (L.L.); yangxq@scsfri.ac.cn (X.Y.); skyxianghuan@163.com (H.X.); 2 Co-Innovation Center of Jiangsu Marine Bio-Industry Technology, Jiangsu Ocean University, Lianyungang 222005, China; 3 College of Food Science & Technology, Shanghai Ocean University, Shanghai 201306, China; 4 Collaborative Innovation Center of Provincial and Ministerial Co-Construction for Marine Food Deep Processing, Dalian 116034, China

**Keywords:** *Grateloupia livida*, hydrolysates, antioxidant peptides, purification, identification

## Abstract

*Grateloupia livida* protein was hydrolyzed with various proteases (alkaline protease, Protamex and neutral protease) to obtain anti-oxidative peptides. Antioxidant activity of the enzymatic hydrolysates was evaluated by the DPPH radical scavenging, ABTS radical scavenging and reducing power assays. The results suggested that hydrolysates obtained by neutral protease 1 h hydrolysis displayed the highest antioxidant activity (DPPH IC_50_ value of 3.96 mg/mL ± 0.41 mg/mL, ABTS IC_50_ value of 0.88 ± 0.13 mg/mL and reducing power of 0.531 ± 0.012 at 8 mg/mL), and had low molecular weight distribution (almost 99% below 3 kDa). Three fractions (F1–F3) were then isolated from the hydrolysates by using semi-preparative RP-HPLC, and the fraction F3 showed the highest antioxidant ability. Four antioxidant peptides were identified as LYEEMKESKVINADK, LEADNVGVVLMGDGR, LIDDSFGTDAPVPERL, and GLDELSEEDRLT from the F3 by LC-MS/MS. Online prediction showed that the four peptides possessed good water solubility, non-toxic and non-allergenic characteristics. Moreover, the LYEEMKESKVINADK exhibited the highest antioxidant ability. Molecular docking revealed that these peptides could all well bind with Kelch-like ECH-associated protein 1 (Keap1), among which LYEEMKESKVINADK had the lowest docking energy (−216.878 kcal/mol). These results demonstrated that the antioxidant peptides from *Grateloupia livida* could potentially be used as natural antioxidant.

## 1. Introduction

Free radicals are very unstable and tend to cause oxidative stress reactions. Excessive free radicals can interact with unsaturated fatty acids, DNA, RNA and other substances in the body, leading to many diseases that are harmful to the body’s health [1]. In addition, the presence of free radicals can adversely affect the flavor and texture of foods. At present, synthetic antioxidants such as propyl gallate (PG), butylhydroxy anisole (BHA) and butylhydroxy toluene (BHT) have been applied in food and cosmetics, which can effectively inhibit the oxidation of products and extend the shelf life of products [2]. However, synthetic antioxidants have toxic and side effects. Using a large amount of synthetic antioxidants will not only damage human organs, but will also affect the stability of products. Therefore, it is very important to find safe and stable natural antioxidants instead of synthetic antioxidants.

In recent years, antioxidant peptides derived from food proteins have become one of the research hotspots of functional peptides. Antioxidant peptides from natural protein sources have the advantages of easy absorption, non-toxicity or low toxicity compared with chemically synthesized drugs, and also have substantial advantages in activity and stability [3,4]. Marine algae-derived proteins are gradually used in the preparation of natural antioxidant peptides [5]. At present, researchers have prepared antioxidant active peptides from a variety of marine algae, such as asparagus [6], spirulina [7] and so on. The peptide AF isolated from the hydrolyzates of *Pyropia columbina* showed the lower IC_50_ value for ABTS free radical (IC_50_ = 0.6 mg/mL) and DPPH free radical (IC_50_ = 1.0 mg/mL) scavenging [8]. The peptides obtained from *Schizochytrium Limacinum* by hydrolysis with compound proteases (Protamex and Alcalase 2.4L) presented the potent DPPH radical scavenging ability (IC_50_ = 1.28 mg/mL) [9].

The growth environment of marine algae is different from that of terrestrial plants, which leads to the differences between specific nutrients and active substances. Moreover, the nutritional value of marine algae was higher than that of terrestrial vegetables, especially in protein composition and amino acid sequence [10]. The structure of antioxidant peptides is simpler than that of their parent proteins, which endows them with greater stability under adverse conditions such as high temperature and protease. They do not cause dangerous immune responses and often exhibit enhanced nutritional and functional properties in addition to antioxidant activity [11]. Therefore, marine algal protein may become an important protein source of new antioxidant peptides.

*Grateloupia livida* (*G. livida*), belonging to Rhodophyta, Rhodophyceae, Gigartinales, Halymeniaceae and Grateloupia, is well known as FoZucai in Guangdong province in China. It is a kind of commercially important red macroalgal species, and widely distributed in the intertidal zones of coastlines (in the South China Sea). Existing studies have proven that the protein of *Grateloupia livida* (*G. livida*) is a good source of natural antioxidants, but the research on the bioactive peptide of *G. livida* has not been reported. The results showed that *G. livida* contained amino acids, proteins, sugars, terpenoids and other bioactive components [12]. In fact, the essential amino acid content (EAA) of *G. livida* is 42.58% of the total amino acid content (TAA), and the amino acid composition is good and balanced. Phycoerythrin from *G. livida* can effectively enhance the antioxidant capacity of primary rat astrocytes [13].

In the present study, alkaline protease, protamex and neutral protease were used to hydrolyze the *G. livida* protein to obtain antioxidant peptides based on the preliminary experiments, and the degree of hydrolysis at different hydrolysis time was determined. The antioxidant activity of enzymatic hydrolysates at different hydrolysis time was evaluated by DPPH radical scavenging activity, ABTS radical scavenging activity and reducing power. Then, the peptides were separated and enriched by reversed-phase high-performance liquid chromatography. The amino acid sequence of the component with high antioxidant activity was analyzed by LC-MS/MS. Combined with the secondary mass spectrometry data by mascot software, four polypeptides were identified. The physicochemical properties (theoretical isoelectric point, hydrophilic average coefficient, water solubility, toxicity and allergenicity) of the four polypeptides were predicted by an online database and analysis tools. Molecular docking was used to further study the combination between the peptides and Keap1. This study could provide a scientific and technical basis for further research on the use of the antioxidant peptides from *G. livida* protein as health products or functional food ingredients.

## 2. Materials and Methods

### 2.1. Materials

*G. livida* was collected from Nan Ao Island (116°56′–117°09′ E and 23°23′–23°29′ N). Epiphytes were removed by hand and samples were dried at 25 °C, followed by hot air-drying for further analysis. Alkaline protease (200 U/mg), Protamex (90 U/mg) and neutral protease (100 U/mg) were purchased from Hefei Bomei Biotechnology Co., Ltd. (Hefei, China). Trifluoroacetic acid (TFA), acetonitrile, methanol, cytochrome C (12,400 Da) and aprotinin (6411.24 Da) were bought from Shanghai Macklin Biochemical Co., Ltd. (Shanghai, China). DPPH and L-oxidized glutathione (612.63 Da) were obtained from Sigma Chemical Co. (St. Louis, MO, USA). Bacitracin (1422.69 Da) and reduced glutathione (307.3 Da) were purchased in Guangzhou Qiyun Biotechnology Co., Ltd. (Guangzhou, China). All other chemicals and reagents used were of analytical grade.

### 2.2. Preparation of G. Livida Protein

*G. livida* was dried in an oven at 60 °C to constant weight, and then crushed to a powder form through a 120-mesh sieve. An appropriate amount of *G. livida* powder was taken, and water was added to it according to the liquid–solid ratio of 160 mL/g. After stirring evenly, ultrasonic treatment was carried out to break the cell wall. Ultrasonic treatment conditions were set as follows: ultrasonic interval time 4 s, ultrasonic occurrence time 4 s, ultrasonic whole time 60 min, and ultrasonic power 1440 W. The crude extract was centrifuged at 3800× *g* at 4 °C for 15 min, and kept the supernatant. Under the ice bath condition, ammonium sulfate was added to the supernatant until the saturation reached 65%. After precipitation for 2 h, centrifugation was carried out. The precipitation was taken for dialysis desalination and freeze-dried to obtain the protein of *G. livida*.

### 2.3. Preparation of G. Livida Protein Hydrolysates

The lyophilized protein powder of *G. livida* was weighted and mixed with ultra-pure water at the liquid–solid ratio of 100 mL/g. The pH was adjusted to the optimal conditions for each protease (alkaline protease, Protamex and neutral protease). The protease was added at the enzyme dosage of 10,000 U/g, and hydrolysis was carried out at the optimal temperature for each protease. During enzymatic hydrolysis, the pH of the system was adjusted once an hour to keep it within the optimal pH ± 0.02 range. After the hydrolysis, the enzyme was inactivated in a water bath at 95 °C for 15 min. The enzyme was centrifuged at 6800× *g* at 4 °C for 15 min after cooling. The supernatant was taken to measure the degree of hydrolysis. 

### 2.4. Determination of Degree of Hydrolysis

The content of amino nitrogen (AN) was determined by automatic potential titration. Briefly, potassium hydrogen phthalate was used to calibrate the actual concentration of 1 mol/L NaOH. Then, hydrolysate (5 mL) was added to deionized water (100 mL) and titrated with 1 mol/L NaOH to the pH value of 8.2. Thereafter, additional formaldehyde solution (5 mL) was fully mixed and titrated with 1 mol/L NaOH to the pH value of 9.2. The volume (mL) of NaOH consumed was recorded as V_1_. The same volume of deionized water was used to take place the hydrolysate for the blank experiment, and the volume (mL) of NaOH consumed was recorded as V_2_. AN was calculated according to the following formulation: AN (g/mL) = (V_1_ − V_2_) × C × 0.014/5, where C is the actual concentration of NaOH (mol/L), 0.014 is the mass (g) of nitrogen equivalent to 1 mL of 1 mol/L NaOH, and 5 is the volume (mL) of sample. The degree of hydrolysis (DH) was calculated as follows:

DH = (AN × V × 6.25)/m × 100%(1)

where AN is the content of amino nitrogen in the supernatant obtained by centrifugation of protein hydrolysate, g/mL; V is the total volume of supernatant obtained by centrifugation of protein hydrolysate, mL; m is the mass of the protein used for hydrolysis, g; and 6.25 is the conversion coefficient of ammonia nitrogen.

### 2.5. Determination of Antioxidant Activity

#### 2.5.1. Determination of Reducing Power

We referred to the experimental method of Oyaizu et al. [14] and slightly modified it; 1 mL phosphate-buffered solution (pH 6.6, 0.2 mol/L), 1 mL sample and 1 mL potassium ferricyanide with a volume fraction of 1% were successively added into a 10 mL centrifuge tube. The mixed reaction solution was placed in a water bath at 50 °C for 20 min. Then, 1 mL trichloroacetic acid with 10% volume fraction was added, and the supernatant was extracted after centrifugation at 10,600× *g* at 4 °C for 10 min. Finally, 1 mL of supernatant was taken, into which 1 mL of deionized water and 0.2 mL of 0.1% ferric chloride solution were added. After vortex mixing, the solution was placed in a water bath at 50 °C and kept for 10 min. The absorbance was measured at 700 nm wavelength. The formula of reducing force is as follows:

Reducing power = AEG − ABG(2)

where AEG is the absorbance in the presence of the hydrolysate and ABG is the absorbance in the presence of 1 mL deionized water.

#### 2.5.2. Determination of DPPH Radical Scavenging Rate

We referred to the experimental method of Boath et al. [15] and made some modifications. The lyophilized enzymatic hydrolysates were prepared into solutions with mass concentrations of 1, 2, 4, 6 and 8 mg/mL, respectively. DPPH was dissolved in anhydrous ethanol and prepared into 0.2 mmol/L solution.1 mL sample and 0.2 mL DPPH solution were successively added into a 10 mL centrifuge tube. The mixed reaction solution was placed in the dark at room temperature for 30 min, and the absorbance was measured at 517 nm wavelength. The sample was replaced by deionized water in the control group, and the DPPH solution was replaced by anhydrous ethanol in the blank group. The calculation formula of DPPH free radical scavenging rate is as follows:

DPPH radical scavenging rate = [1 − (A_1_ − A_2_)/A_0_] × 100% (3)

where A_0_ is the absorbances of the control group, A_1_ is the absorbances of the experimental group, and A_2_ is the absorbances of the blank group.

#### 2.5.3. Determination of ABTS Radical Scavenging Rate

The ABTS radical scavenging rate was determined according to the former report [16] with some slight modification. A methanol solution of 50% was used to dissolve ABTS and prepare a 7 mmol/L solution. The solution was mixed 1:1 with 2.45 mmol/L potassium persulfate solution and placed in the dark at room temperature for 16 h to obtain ABTS reserve solution. The ABTS working fluid was obtained by diluting the reserve solution to an absorbance of 0.70 ± 0.02 at 734 nm before use. Successively, 1 mL sample solution and 4 mL ABTS working solution were added into a 10 mL centrifuge tube, and the mixed reaction solution was reacted for 10 min away from light. The absorbance was measured at the wavelength of 734 nm. The ABTS working fluid was replaced by 50% methanol solution and deionized water in the control group and the blank group, respectively. The calculation formula of ABTS free radical scavenging rate is as follows:

ABTS radical scavenging rate = [1 − (A_1_ − A_2_)/A_0_] × 100% (4)

where A_0_ is the absorbances of the blank group; A_1_ is the absorbances of the experimental group; and A_2_ is the absorbances of the control group.

### 2.6. High-Performance Size Exclusion Chromatography

With reference to the method of Gendis et al. [17], the desalted enzymatic hydrolysate was dissolved in ultra-pure water and prepared into a solution of 2 mg/mL. The solution was filtered with 0.22 μm filter membrane, and its molecular weight was determined by high-performance size exclusion chromatography (LC-20AD, Shimadzu Co., Tokyo, Japan). The chromatographic conditions were as follows: HPLC column TSK-GEL G2000SWXL (7.8 mm × 300 mm, Tosoh Co., Tokyo, Japan); detection wavelength was 214 nm; mobile phase A was 0.1% TFA aqueous solution, and mobile phase B was acetonitrile containing 0.1%TFA (A:B = 80:20); injection volume was 10μL; flow rate was 0.5 mL/min, and the total elution time was 40 min. Cytochrome C (12,400 Da), aprotinin (6411.24 Da), bacitracin (1422.69 Da), L-oxidized glutathione (612.63 Da) and reduced glutathione (307.3 Da) were chosen for molecular weight standards. The above standards were dissolved in mobile phase B, quality of mixture concentration is 0.2 mg/mL of mixed standard solution. The lyophilized powder of the enzymatic hydrolysate was prepared into a solution of 2 mg/mL with ultra-pure water, and the samples were injected under the same conditions for analysis.

### 2.7. Reversed-Phase High-Performance Liquid Chromatography (RP-HPLC)

According to the method of Sun et al. [18] with some modifications, the desalted hydrolysates were collected and filtered with 0.22 μm microporous membrane. The products were further separated and purified by RP-HPLC. The peptide fraction was filtered with a membrane (0.22 μm) before it was loaded into ZORBAX XDB-C18 (21.2 × 150 mm, 5 μm). The mobile phases used in the gradient elution consisted of eluent A consisting of ultrapure water (*v*/*v*) and eluent B consisting of methanol. The column was eluted with linear gradient from 1 to 1% mobile phase B from 0 to 3 min, 1 to 30% mobile phase B from 3 to 8 min, 30 to 80% mobile phase B from 8 to 18 min, 80 to 40% mobile phase B from 18 to 23 min, 40 to 10% mobile phase B from 23 to 28 min, 10 to 1% mobile phase B from 28 to 33 min and 1 to 1% mobile phase B from 33 to 38 min at 3 mL/min. The eluate was monitored at 268 nm. Each elution peak was collected for freeze-drying, and the components with strong antioxidant activity were screened out with the index of reducing power and IC_50_ of DPPH radical scavenging rate.

### 2.8. Identification of the Amino Acid Sequence by LC-MS/MS

*G. livida* antioxidant peptide was further analyzed regarding the amino acid sequence and its accurate molecular weight by LC-MS/MS on Q Exactive Plus mass spectrometer. The peptide solution was desalted and redissolved by Empore™ SPE Cartridges C18 (Standard density), and the sample was injected into Acclaim PepMap C18 column (75 μm × 150 mm) at a flow rate of 300 nL/min. The mobile phases used in the gradient elution consisted of 0.1% formic acid in water (mobile phases A) and 0.1% formic acid in CAN (mobile phases B). The column was eluted with linear gradient from 0 to 5% mobile phases B from 0 to 5 min, 5 to 50% mobile phases B from 5 to 45 min, 50 to 90% mobile phases B from 45 to 55 min, and 90 to 5% mobile phases B from 55 to 65 min. The mass spectrometer was run under data-dependent acquisition mode, and automatically switched between MS and MS/MS mode. The parameters were as follows: (1) MS: scan range (m/z) = 100–2000; resolution = 70,000; AGC target = 3e6; maximum injection time = 40 ms; (2) HCD-MS/MS: resolution = 17, 500; isolation window = 2.0; AGC target = 1e5; maximum injection time = 60 ms; collision energy = 28. Tandem mass spectra were processed by MM File Conversion. The amino acid sequence and its source can be obtained by searching in the UniProt database (Rhodymeniophycidae: https://www.uniprot.org/taxonomy/2045261) (accessed on 16 November 2021).

### 2.9. Property Prediction of the Identified Peptides and Their Antioxidant Ability 

The parameters of physical and chemical properties of the identified peptides (theoretical isoelectric point (PI), hydrophilic average coefficient (GRAY), water solubility, toxicity and allergenicity) were evaluated in silico. The PI and GRAY of the peptides were determined by the ProtParam tool on Expasy server (https://web.expasy.org/protparam) (accessed on 20 December 2021). The water solubility of the peptides was determined by Innovagen tool (http://www.innovagen.com/proteomics-tools) (accessed on 20 December 2021). Toxicity of the peptides was forecasted by ToxinPred tool (https://webs.iiitd.edu.in/raghava/toxinpred/multi_submit.php) (accessed on 20 December 2021). The allergenicity of the peptides was predicted by AllerTOP tool (https://www.ddg-pharmfac.net/AllerTOP/index.html) (accessed on 20 December 2021). The identified peptides were synthesized by GL Biochem (Shanghai, China) Ltd. for the evaluation of their antioxidant activity, and the antioxidant ability was determined according to 2.5.2 with the concentration at 0.5 mg/mL.

### 2.10. Molecular Docking Analysis

The X-ray crystal structure of keap1 (PDB ID: 2FLU) was obtained from the RCSB Protein Data Bank (https://www.rcsb.org/) (accessed on 18 February 2022). All the water molecules and the chain B (Nrf2) in Kelch-like ECH-associated protein 1 (keap1) were removed sequentially by Pymol software, followed by adding the hydrogens. Then, the modified keap1 and target peptide sequence were employed for docking simulation study using online docking server (http://huanglab.phys.hust.edu.cn/hpepdock/) (accessed on 18 February 2022) and the best conformation was downloaded. Finally, Pymol software was used to illustrate the interaction of the peptides of the best conformation with modified keap1 by molecular docking.

### 2.11. Statistical Analysis

The assay for the samples was conducted in triplicates, and the results were expressed as mean ± standard deviation. SPSS 22.0 software (IBM Corp., Armonk, NY, USA) was used for data analysis. One-way analysis of variance (ANOVA, Tukey test) was used for significance test (*p* < 0.05 was considered a significant difference).

## 3. Results and Discussion

### 3.1. DH and Antioxidant Activity of the Protein Enzymatic Hydrolysates

**Figure 1:**
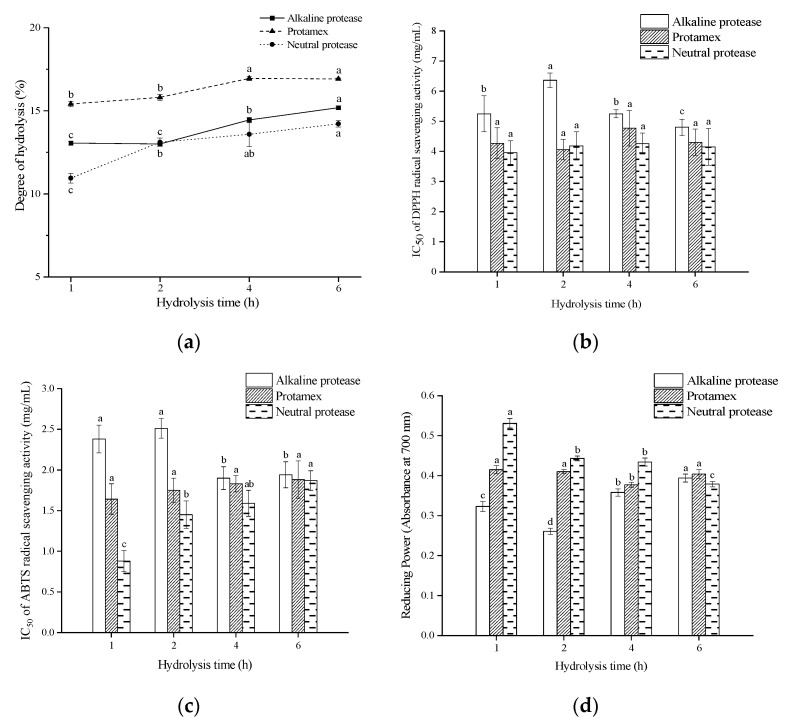
The DH and antioxidant activity of different hydrolysates under different hydrolysis times. (**a**) DH; (**b**) IC_50_ of the hydrolysates for DPPH radical scavenging; (**c**) IC_50_ of the hydrolysates for ABTS radical scavenging; (**d**) Reducing power of the hydrolysates at 8 mg/mL. Different letters on the top of the same pattern (point or column) indicate the significant difference between the hydrolysates obtained by the same protease at different hydrolysis times (*p* < 0.05).

The degree of hydrolysis (DH) can directly reflect the breaking degree of protein peptide bonds, and can be used as an important index to evaluate the extent of the enzymatic degradation [19]. Each protease has a specific cleavage site and action conditions. When applied to the hydrolysis of the same protein, the degree of hydrolysis will be different, which will also lead to the difference in the biological activity of the product. Najafian et al. [20] obtained the antioxidant peptides from patin (*Pangasius sutchi*) myofibrillar protein by hydrolysis, and found that the change trend of the antioxidant activity of the hydrolysate was similar to that of the DH. Irshad et al. [21] prepared the antioxidant peptides from bovine casein by hydrolysis, and found that the free radical scavenging activity of the hydrolysate was positively correlated with the DH. Therefore, it is necessary to study the degree of hydrolysis of protein under different conditions. As shown in Figure 1a, the DH of all the hydrolysates was in a rising trend with the extension of enzymatic hydrolysis time. Moreover, the DH of the hydrolysate prepared by Protamex was obviously higher than that of the hydrolysate prepared by alkaline protease and neutral protease.

The antioxidant activity of the peptides is not only related to the chain length, but also related to the composition of amino acids, amino acid sequence, side chain and spatial conformation [22]. Therefore, in addition to analyzing the DH, it is still necessary to verify the actual antioxidant activity of the enzymatic hydrolysates. The antioxidant activities of peptides can be evaluated by determining their radical scavenging activity and reducing power. The IC_50_ of radical (DPPH and ABTS radical) scavenging activity and reducing power of the hydrolysates are shown in Figure 1b–d. It was found that there was a large difference in the antioxidant activity of the hydrolysates prepared under the different hydrolysis time. This may be due to the different chain length, molecular weight, amino acids composition and sequence of the peptides obtianed from *G. livida* at different enzymatic hydrolysis times, leading to different exposure level of special groups reacting with free radicals, and finally resulting in differences in antioxidant activity of the enzymatic hydrolysates. It was also found that the antioxidant activity of the hydrolysates prepared by neutral protease (1 h hydrolysis) showed the highest antioxidant activities, which presented strong DPPH radical scavenging activity (IC_50_ = 3.96 ± 0.41 mg/mL), ABTS radical scavenging activity (IC_50_ = 0.88 ± 0.13 mg/mL), and reducing power (0.531 ± 0.012 at 8 mg/mL). Additionally, the antioxidant activities of the hydrolysates prepared by different proteases were all stronger than that of unhydrolyzed *G. livida* protein (IC_50_ of ABTS radical scavenging rate was 3.96 ± 0.99 mg/mL), suggesting that the hydrolysis was beneficial for improving the antioxidant activity of the *G. livida* protein. After the *G. livida* protein was hydrolyzed by neutral protease (1 h hydrolysis), the protein yield was 78.12%, and the DPPH radical scavenging activity of the hydrolysate was obviously lower than that of the GSH (IC_50_ = 0.16 ± 0.08 mg/mL) because the obtained hydrolysate was a complex mixture of various peptides. Finally, the *G. livida* protein hydrolysate prepared by neutral protease (1 h hydrolysis) was selected for separation and purification in order to obtain highly active antioxidant peptides.

### 3.2. Molecular Weight Distribution of the Enzymatic Hydrolysate

It is well known that the components with higher molecular weight have shorter retention time in high performance size exclusion chromatography (HPSEC). The molecular weight (MW) distribution of the hydrolysate with high antioxidant activity was determined by HPSEC, using the retention time (min) as the horizontal coordinate (x) axis and the lg MW of the standard substance as the vertical coordinate (y) axis to draw the standard curve. The linear regression equation of the standard curve was y = −0.1802x + 6.5707, R^2^ = 0.9902.

**Figure 2:**
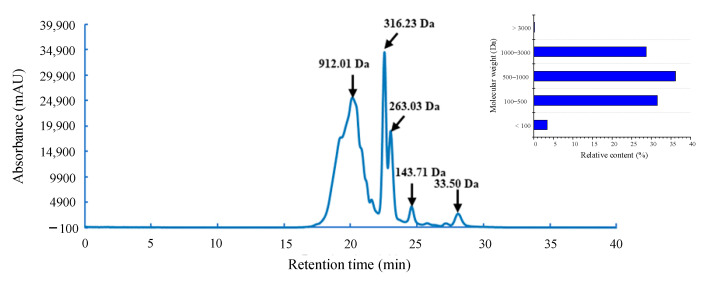
Molecular weight distribution of *G. livida* protein hydrolysate prepared by neutral protease for 1 h hydrolysis.

As a previous study noted, peptides within the appropriate MW range can exert free radical scavenging effects through hydrogen donor groups [23]. Figure 2 shows the MW distribution of the *G. livida* protein hydrolysate prepared by neutral protease (1 h hydrolysis). It can be seen that after hydrolysis of *G. livida* protein by neutral protease for 1 h, the MW of the resultant peptides was relatively small, and the MW of more than 99% of the peptides in the hydrolysate was below 3000 Da. Among them, there were about 28.69% of the peptides in the MW range of 1000–3000 Da, about 36.14% of the peptides in the MW range of 500–1000 Da, and about 34.96% of the peptides with the MW below 500 Da.

### 3.3. Semi-Preparative RP-HPLC Purification

The antioxidant peptides can be purified by the reversed-phase high-performance liquid chromatography (RP-HPLC) based on the polarity [24]. In the present study, the *G. livida* protein hydrolysate with high antioxidant activity was further separated and purified by semi-preparative RP-HPLC. 

**Figure 3:**
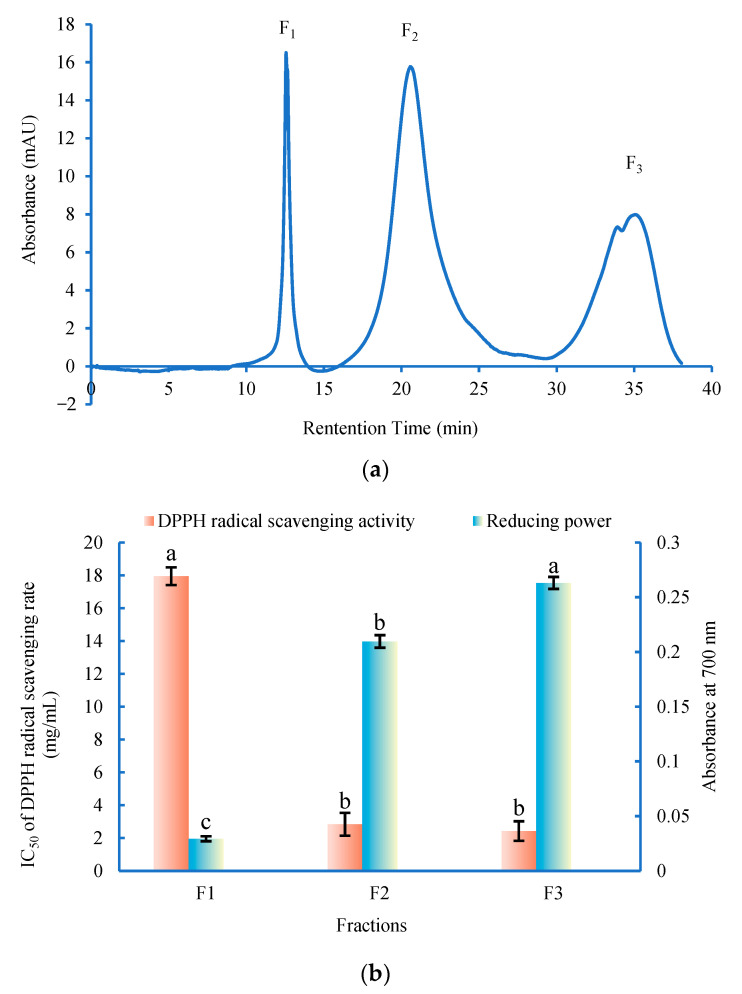
Separation of *G. livida* hydrolysate by semi-preparative RP-HPLC and the antioxidant activities of the peptide fractions. (**a**) Semi-preparative RP-HPLC; (**b**) The reducing power (at 4 mg/mL) and the IC_50_ of DPPH radical scavenging rate of the fractions. Different letters on the top of the same pattern column indicate the significant difference between the fractions in the same test (*p* < 0.05).

As shown in Figure 3a, the elution was separated into three fractions (F1, F2 and F3). The antioxidant activity of the fractions was shown in Figure 3b. Among these three fractions, the fraction F3 (protein yield was 25.28%) exhibited the strongest antioxidant activity (the IC_50_ of DPPH radical scavenging rate was 2.43 ± 0.59 mg/mL and the reducing power was 0.263 ± 0.005 at 4 mg/mL), which was also obviously higher than that of the crude hydrolysate. It has been proven that the eluting order of each fraction peak in the RP-HPLC is correlated to its polarity. According to the linear gradient elution, the fraction F3 eluted within a relatively longer retention time, suggesting that fraction F3 had high antioxidant activity might be due to the high content of polar amino acids. The structural characteristics of the antioxidant peptides in the fraction F3 were further identified.

### 3.4. Identification of Antioxidant Peptide by LC-MS/MS

Antioxidant activity of peptide is considered to be related to its molecular weight, amino acid composition and sequence. The molecular weight and amino acid sequence of the purified antioxidant peptides (fraction F3) were determined by liquid chromatography tandem mass spectrometry (LC-MS/MS). 

**Figure 4:**
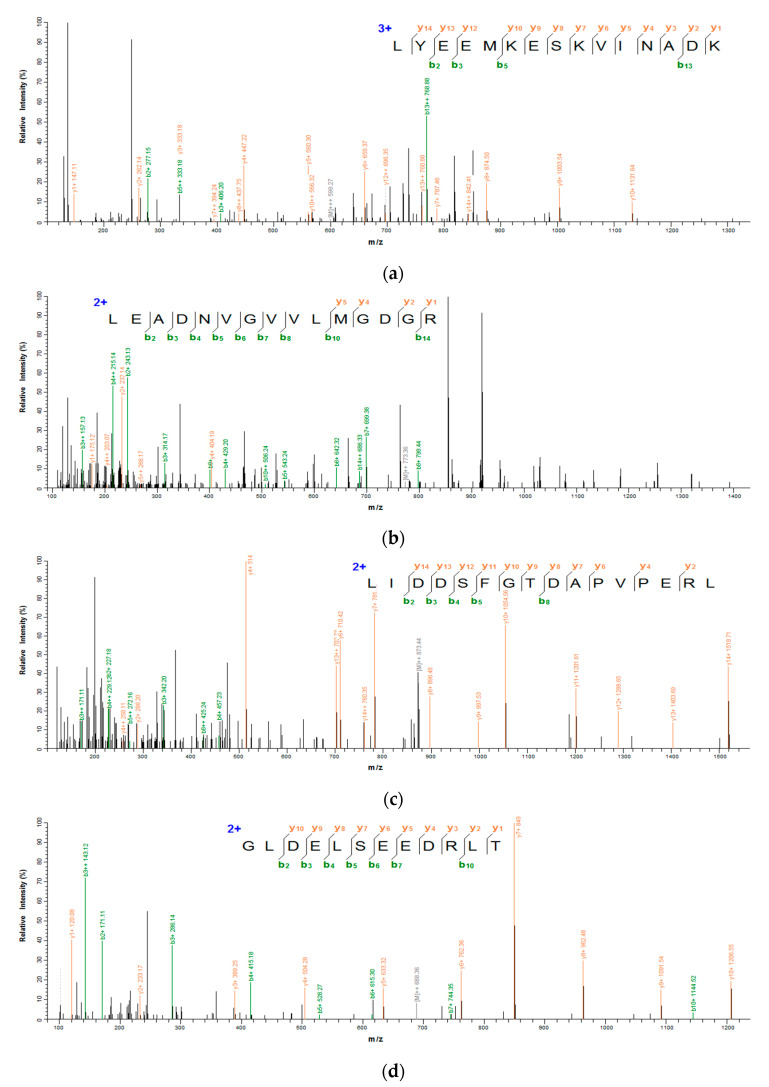
Secondary mass spectrum of antioxidant peptides from *G. livida*. (**a**) LYEEMKESKVINA DK; (**b**) LEADNVGVVLMGDGR; (**c**) LIDDSFGTDAPVPERL; (**d**) GLDELSEEDRLT.

Figure 4a–d show the secondary mass spectrums of the antioxidant peptides. The secondary mass spectra of y and b ions in each peptide had a high degree of matching with the ions in mascot database, indicating that the identification results were accurate. On the basis of LC–MS/MS analysis and database retrieval, four sequences with 12–16 amino acid residues and the molecular weight of the peptides are shown in Table 1. The molecular weight of each peptide was in the range of 1376.72–1797.81 Da, and the theoretical molecular weight of these peptides was consistent with the actual molecular weight. Moreover, the molecular weight of antioxidant peptides was similar to that of VKAGFAWTANQQLS (1519 Da) from tuna [25] as well as that of LEEQQQTEDEQQDQL (1860.85 Da) and YLEELHRLNAGY (1477.63 Da) from camel milk [26], which was in accordance with the molecular weight range of food derived antioxidant peptides (usually from 500 to 1800 Da).

### 3.5. Physical and Chemical Properties of the Identified Peptides

As shown in Table 2, the physical and chemical properties (PI, GRAY, water solubility, toxicity and allergenicity) of the identified peptides were evaluated in silico. The PI and GRAY of the peptides were determined by the Protparam tool on Expasy server. It was found that the PI of these four peptides was from pH 3.83 to pH 4.87 due to the high content of acidic amino acid residues, and most of them had good hydrophily (GRAVY < 0). The water solubility of the peptides was estimated by Innovagen tool, and the result suggested that all these peptides had good water solubility. In order to further characterize the potential of the antioxidant peptides from *G. livida* as functional ingredients in food or beverage, it is necessary to evaluate their potential toxicity and allergenicity [27]. The toxicity and allergenicity of the peptides were predicted by ToxinPred tool and AllerTOP tool, respectively. The analysis indicated that all the identified peptides were non-toxin and non-allergen. 

Finally, the identified peptides were synthesized, and the IC_50_ of DPPH radical scavenging activity of the peptides was investigated. It can be seen that the four peptides possessed different DPPH radical scavenging activity with the order of LYEEMKESKVINADK > LEADNVGVVLMGDGR > LIDDSFGTDAPVPERL > GLDELSEEDRLT. The former research claimed that the peptides which were rich in hydrophobic amino acids and acidic acids could present higher antioxidant ability, and also that the repetitive di- or tri-amino acid residues within a peptide could be related to the high antioxidant activity of peptide [28]. For the four identified peptides, all of them had hydrophobic amino acids, acidic acids and repetitive di-amino acid residues. In addition, the fact that the LYEEMKESKVINADK had higher DPPH radical scavenging activity might be attributed to the two fragments of LY and KVI, which could enhance the antioxidant ability according to the calculation results in the BIOPEP database (https://biochemia.uwm.edu.pl/biopep-uwm/) (accessed on 18 February 2022). The amino acid sequence and the presence of certain amino acid residues (such as H, Y, W, F, C, and K) have also been shown to be determining factors in the antioxidant capacity of peptides (designing antioxidant peptides based on the antioxidant properties of the amino acid side-chains), and V or L at N-terminus in peptides could exhibit higher antioxidant ability. In the four peptides, only the LYEEMKESKVINADK possesses Y and K in the sequence, and also has the L at N-terminus, which was in accordance with the result that LYEEMKESKVINADK had higher DPPH radical scavenging activity. 

### 3.6. Molecular Docking Results of the Identified Peptides

Nuclear factor erythroid-2 related factor 2 (Nrf2) principally modulates endogenous defense in response to oxidative stress in the body, which was negatively regulated by Kelch-like ECH-associated protein 1 (Keap1) [29]. In common conditions, Keap1 can bind to Nrf2 and result in the degradation of Nrf2. When cells are subjected to oxidative stress, Nrf2 can activate the antioxidant enzyme system to protect the cells [30]. Nrf2 has been considered as a good transcription factor that protects the body from many diseases. Hence, the molecules (such as peptides) that can bind to Keap1 would inhibit the combination between Keap1 and Nrf2, which is beneficial for suppressing oxidative stress in the body’s cells. 

**Figure 5:**
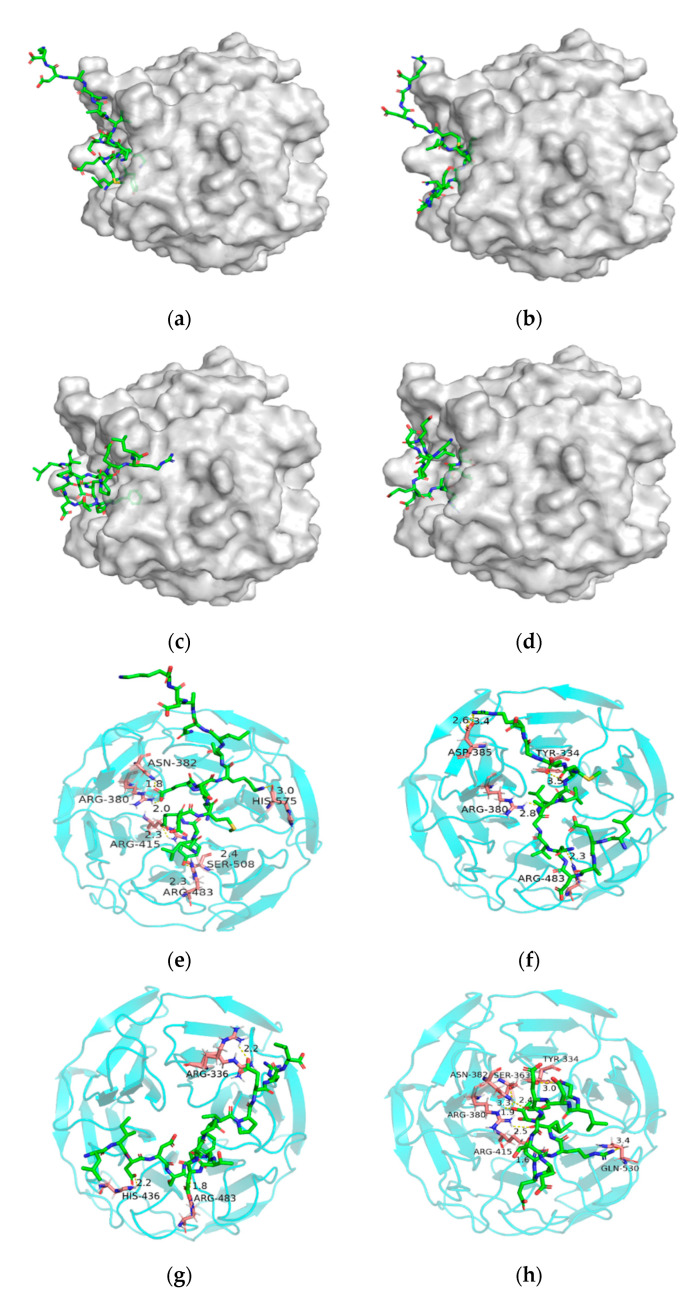
Molecular docking results of LYEEMKESKVINADK (**a**,**e**), LEADNVGVVLMGDGR (**b**,**f**), LIDDSFGTDAPVPERL (**c**,**g**), GLDELSEEDRLT (**d**,**h**). (**a**–**d**) reflect the surface docking simulations between the peptides and Keap1, and (**e**–**h**) reflect the peptides binding with amino acid residues in the Keap1 activity center.

As shown in Figure 5a,e, LYEEMKESKVINADK formed six hydrogen bonds with ARG415, ARG380, ASN382, HIS575, SER508 and ARG483 of Keap1 with docking energy of −216.878 kcal/mol. As shown in Figure 5b,f, LEADNVGVVLMGDGR formed four hydrogen bonds with ASP385, TYR334, ARG380 and ARG483 of Keap1 with a docking energy of −186.917 kcal/mol. In addition, LIDDSFGTDAPVPERL formed three hydrogen bonds with ARG483, HIS436 and ARG336 of Keap1 with a docking energy of −207.665 kcal/mol (Figure 5c,g). Moreover, GLDELSEEDRLT formed six hydrogen bonds with TYR334, SER363, ARG380, ASN382, ARG415 and GLN530 with a docking energy of −171.876 kcal/mol (Figure 5d,h). The results suggested that LYEEMKESKVINADK combined with Keap1 was more easy than the other three peptides due to the lowest docking energy, which was in accord with its higher antioxidant activity.

It was also found that ARG380, ARG483, ARG415, ASN382 and TYR334 in the Keap1 were the main binding sites for the peptides according to the docking results. This might be attributed to the functional groups such as guanidine group of ARG and aromatic nucleus of TYR. The previous study also reported the importance of these amino acid residues in conjugation [31]. It was suggested that the antioxidant peptides from *G. livida* might have a good protection on the cells in terms of suppressing the oxidative stress.

## 4. Conclusions

In the present study, *Grateloupia livida* hydrolysates were obtained by hydrolysis with three proteases (alkaline protease, Protamex and neutral protease), respectively. Under neutral protease treatment (1 h), the hydrolysates showed the highest antioxidant ability and low molecular weight distribution (almost 99% below 3 kDa). Three fractions (F1–F3) in the hydrolysates were then screened by using semi-preparative RP-HPLC, and four antioxidant peptides were identified from the fraction (F3) with the highest antioxidant activity by LC-MS/MS. Their amino acid sequences were verified as LYEEMKESKVINADK, LEADNVGVVLMGDGR, LIDDSFGTDAPVPERL and GLDELSEEDRLT, respectively. Online prediction demonstrated that the four peptides possessed good water solubility, and non-toxic and non-allergenic characteristics. In addition, it was found that the DPPH radical scavenging activity of the LYEEMKESKVINADK was higher than that of the other peptides. Molecular docking revealed that these peptides could all well bind with Keap1, among which LYEEMKESKVINADK showed the lowest docking energy, suggesting that it was more easily combined with Keap1. The present results indicated that the antioxidant peptides from *Grateloupia livida* hydrolysates could be potentially used as an ingredient in new functional foods. Further research work needs to be conducted on providing more useful information regarding in vivo antioxidant activity to identify practical applications for those peptides.

## Figures and Tables

**Table 1:** Antioxidant peptides from *G. livida* identified by LC–MS/MS.

Pepetide Sequence	Molecular Weight (Da)	Structure Formula
LYEEMKESKVINADK	1797.81	* 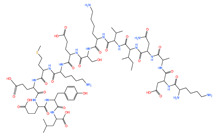 *
LEADNVGVVLMGDGR	1546.72	* 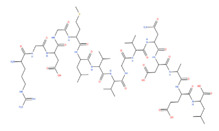 *
LIDDSFGTDAPVPERL	1746.88	* 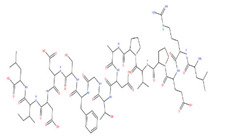 *
GLDELSEEDRLT	1376.72	* 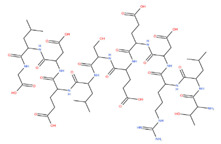 *

**Table 2:** Physical and chemical properties of antioxidant peptides from *G. livida*.

Pepetide Sequence	pI	GRAVY	Water Solubility	Toxicity	Allergenicity	DPPH Radical Scavenging Activity (IC_50_, mg/mL)
LYEEMKESKVINADK	4.87	−1.007	Good	non-toxin	Probable non-allergen	0.53 ± 0.04 ^c^
LEADNVGVVLMGDGR	4.03	0.28	Good	non-toxin	Probable non-allergen	0.69 ± 0.06 ^b^
LIDDSFGTDAPVPERL	3.84	−0.169	Good	non-toxin	Probable non-allergen	0.72 ± 0.04 ^b^
GLDELSEEDRLT	3.83	−1.042	Good	non-toxin	Probable non-allergen	1.07 ± 0.09 ^a^

Values within the same column followed by the same letter are not significantly different (*p* > 0.05).

## Data Availability

Data are contained within the article.
